# Digital eye tracking and plasma biomarkers: Distinguishing functional cognitive impairment from Alzheimer's disease biology

**DOI:** 10.1002/alz.71575

**Published:** 2026-06-09

**Authors:** Julián Benito‐León, Carla María Benito‐Rodríguez

**Affiliations:** ^1^ Department of Neurology 12 de Octubre University Hospital Madrid Spain; ^2^ Group of Neurodegenerative Diseases Hospital Universitario 12 de Octubre Research Institute (imas12) Madrid Spain; ^3^ Network Center for Biomedical Research in Neurodegenerative Diseases (CIBERNED) Madrid Spain; ^4^ Department of Medicine Faculty of Medicine, Complutense University of Madrid Madrid Spain; ^5^ Department of Neurology Torrejón University Hospital Torrejón de Ardoz, Madrid Spain

1

Dear Editor,

We read with great interest the article by Ling et al.,^1^ “A novel eye‐tracking digital marker outperforms plasma biomarkers in detecting cognitive impairment.” The authors should be congratulated for developing a rapid, engaging, and scalable eye‐tracking paradigm and for integrating digital markers with plasma biomarkers, positron emission tomography, magnetic resonance imaging, and cognitive outcomes. Their finding that an eye‐panel marker classified cognitively impaired individuals more accurately than several plasma biomarkers is provocative and clinically relevant.^1^ However, we believe that the interpretation that eye tracking “outperforms plasma biomarkers” requires important qualification.

The key issue concerns the nature of the diagnostic target. Ling et al.^1^ classified participants as cognitively unimpaired or cognitively impaired (CI), with the latter group combining mild cognitive impairment and dementia.^1^ The eye‐panel marker was derived from a task that included oculomotor measures, instantaneous and delayed recall scores, task‐completion variables, fixation measures, and game‐navigation metrics.^1^ Thus, at least part of the predictor set was a brief cognitive‐performance assessment, whereas the outcome was a clinical cognitive‐status label. This creates substantial criterion proximity: a marker that directly samples memory, processing speed, attention, and task execution is likely to predict a cognitive syndrome better than molecular biomarkers designed to detect Alzheimer's disease (AD) pathology.

This distinction is not merely semantic. Current Alzheimer's Association criteria define AD biologically, not by clinical syndrome alone, and emphasize diagnosis through abnormalities in core biomarkers.^2^ Blood‐based biomarkers such as amyloid‐beta 42/40 (Aβ42/40), phosphorylated tau at threonine 217 (*p*‐tau217), glial fibrillary acidic protein (GFAP), and neurofilament light chain (NfL) are intended to support the evaluation of underlying pathology and neurodegeneration, not to replace cognitive‐performance testing.^3^, ^4^ Therefore, a more precise conclusion would be that Ling et al.’s^1^ eye‐panel marker outperformed plasma biomarkers for the syndromic detection of cognitive impairment, but not necessarily for the detection of AD biology.

The heterogeneous composition of the CI group further complicates comparison. If the CI group included non‐AD, mixed, vascular, inflammatory, or other neurodegenerative processes, AD‐specific plasma biomarkers, particularly Aβ42/40 and *p*‐tau217, would be at a disadvantage. Conversely, digital eye‐tracking measures may capture downstream network dysfunction across etiologies. The relatively strong performance of GFAP and NfL is consistent with this interpretation, as these biomarkers index common pathways of glial reactivity and neuroaxonal damage across diverse neurodegenerative and non‐neurodegenerative conditions, rather than AD–specific amyloid and tau pathology ^1^, ^3^, ^4^

Our experience with eye tracking supports its value as a sensitive functional biomarker and illustrates its transdiagnostic nature. In long coronavirus disease 2019 (COVID‐19), our group demonstrated that eye‐tracking measures combined with machine‐learning approaches can identify cognitive dysfunction and provide evidence of frontal–subcortical circuit involvement.^5^ In survivors of toxic oil syndrome, we found selective antisaccade impairment and executive dysfunction more than four decades after exposure, supporting eye tracking as a noninvasive marker of latent frontal–subcortical dysfunction.^6^ We have also reviewed the expanding role of blood‐based biomarkers in AD diagnosis and prognosis, underscoring the need to distinguish functional cognitive markers from molecular disease markers.^4^

Several additional analyses would strengthen the authors’ conclusions. First, the diagnostic performance of purely oculomotor variables should be reported after excluding recall scores and other task‐performance variables. Second, the eye‐panel should be tested using nested cross‐validation or, preferably, in an independent external cohort, because feature selection and estimation of the area under the receiver operating characteristic curve within the same dataset can yield optimistic estimates. Third, models should report incremental value over age, education, sex, visual acuity, ocular disease, parkinsonism, depression, anxiety, fatigue, sedating medications, Mini‐Mental State Examination, Montreal Cognitive Assessment, and manual‐controller performance. Finally, analyses stratified by amyloid and tau status, such as amyloid‐positive/tau‐positive (A+T+) versus amyloid‐negative/tau‐negative, or AD versus non‐AD cognitive impairment, would clarify whether the eye‐panel detects AD biology, downstream cognitive impairment, or both.

Ling et al.^1^ have made an important contribution by showing that digital eye tracking may provide a scalable and patient‐friendly approach to screening for cognitive impairment. The central message, however, should be refined: eye tracking may outperform plasma biomarkers for classifying a clinical cognitive syndrome, but whether it outperforms, or more likely complements, plasma biomarkers for detecting AD pathology remains to be demonstrated.

## CONFLICT OF INTEREST STATEMENT

The authors declare no conflicts of interest. Author disclosures are available in the Supporting Information.

## ETHICAL APPROVAL

The authors confirm that they have read the Journal's position on ethical publication and affirm that this work is consistent with those guidelines.

## DISCLOSURE

The authors declare no additional disclosures.

## Supporting information




**Supporting Information**: alz71575‐sup‐0001‐ICMJE.pdf
